# What Determines Vulnerability to Neighborhood Walkability in Older Adults?

**DOI:** 10.1093/geroni/igab046.2160

**Published:** 2021-12-17

**Authors:** Andrea Rosso, Caterina Rosano, Alyson Harding, Stephanie Studenski, Philippa Clarke

**Affiliations:** 1 University of Pittsburgh, Pittsburgh, Pennsylvania, United States; 2 University of Minnesota, Minneapolis, Minnesota, United States; 3 Institute for Social Research, Ann Arbor, Michigan, United States

## Abstract

Environmental influences are recognized as important predictors of walking behaviors in older adults. However, individuals may differ in vulnerability to low environmental walkability. We determined associations of a walkability index (factor analysis of 16 variables; range -1.65 to 2.23) from audits of online images with self-reported walking behaviors in 406 adults mean age=82 (44% male, 39% Black). Effect modification by 12 variables representing sociodemographics, physical and mental health, and neighborhood characteristics was tested in general linear models. Effect modification was evident for knee pain, marital status, and neighborhood socioeconomic status (nSES) (all p-interaction<0.05); associations were present only in those with knee pain, those who were unmarried, and those in the highest race-specific tertile of nSES. For example, a 1 point higher walkability score was associated with 1.06 (CI: 0.78, 1.44) higher odds of walking in those without knee pain compared to 1.91 (CI: 1.25, 2.90) in those with knee pain.

